# Characterization of metallothionein genes from *Broussonetia papyrifera*: metal binding and heavy metal tolerance mechanisms

**DOI:** 10.1186/s12864-024-10477-x

**Published:** 2024-06-05

**Authors:** Zhenggang Xu, Shen Yang, Chenhao Li, Muhong Xie, Yi He, Sisi Chen, Yan Tang, Dapei Li, Tianyu Wang, Guiyan Yang

**Affiliations:** 1 College of Forestry, Northwest A & F University, Yangling, 712100 Shaanxi China; 2 Labortory of Walnut Research Center, College of Forestry, Northwest A & F University, Yangling, 712100 Shaanxi China

**Keywords:** Paper mulberry, Metallothionein, Expression analysis, Yeast transformation, Site-directed mutagenesis, Heavy metal transfer

## Abstract

**Background:**

*Broussonetia papyrifera* is an economically significant tree with high utilization value, yet its cultivation is often constrained by soil contamination with heavy metals (HMs). Effective scientific cultivation management, which enhances the yield and quality of *B. papyrifera*, necessitates an understanding of its regulatory mechanisms in response to HM stress.

**Results:**

Twelve *Metallothionein* (*MT*) genes were identified in *B. papyrifera*. Their open reading frames ranged from 186 to 372 bp, encoding proteins of 61 to 123 amino acids with molecular weights between 15,473.77 and 29,546.96 Da, and theoretical isoelectric points from 5.24 to 5.32. Phylogenetic analysis classified these BpMTs into three subclasses: MT1, MT2, and MT3, with MT2 containing seven members and MT3 only one. The expression of most *BpMT* genes was inducible by Cd, Mn, Cu, Zn, and abscisic acid (ABA) treatments, particularly *BpMT2e*, *BpMT2d*, *BpMT2c*, and *BpMT1c*, which showed significant responses and warrant further study. Yeast cells expressing these *BpMT* genes exhibited enhanced tolerance to Cd, Mn, Cu, and Zn stresses compared to control cells. Yeasts harboring *BpMT1c*, *BpMT2e*, and *BpMT2d* demonstrated higher accumulation of Cd, Cu, Mn, and Zn, suggesting a chelation and binding capacity of *BpMT*s towards HMs. Site-directed mutagenesis of cysteine (Cys) residues indicated that mutations in the C domain of type 1 BpMT led to increased sensitivity to HMs and reduced HM accumulation in yeast cells; While in type 2 BpMTs, the contribution of N and C domain to HMs’ chelation possibly corelated to the quantity of Cys residues.

**Conclusion:**

The *BpMT* genes are crucial in responding to diverse HM stresses and are involved in ABA signaling. The Cys-rich domains of *BpMT*s are pivotal for HM tolerance and chelation. This study offers new insights into the structure-function relationships and metal-binding capabilities of type-1 and − 2 plant MTs, enhancing our understanding of their roles in plant adaptation to HM stresses.

**Supplementary Information:**

The online version contains supplementary material available at 10.1186/s12864-024-10477-x.

## Background

Paper mulberry (*Broussonetia papyrifera*) is an economically significant tree species with diverse applications. It is used for high-quality pig feed, its phloem fibers are premium raw materials for papermaking, its roots and seeds have medicinal properties, and its sap can treat skin diseases [1, 2]. Additionally, it contains higher levels of phenolic substances, such as flavonoids, polyphenols, and fructose, compared to many other plants [1, 2], offering substantial potential for development and utilization. To realize the economic potential of *B. papyrifera*, significant cultivation is essential. Given the constraints of not competing with agricultural land, the expansion of *B. papyrifera* cultivation has focused on utilizing less favorable sites such as construction wastelands, riverbanks, and tailings areas. Over the past 10 to 20 years, the cultivation of paper mulberry on heavy metal (HM)-polluted sites has increased rapidly. However, the yields from these sites have generally been much lower than expected. The primary cause is often unscientific cultivation practices in suboptimal soil conditions, including HM pollution and salt retention, despite *B. papyrifera*’s role as a pioneer species in ecological restoration. To optimize cultivation, it is critical to understand the response mechanisms of paper mulberry to various stressors, particularly HMs.

Plants have developed sophisticated strategies to mitigate damage under adverse conditions, including releasing stress signals, adapting to stress stimuli, regulating gene expression, altering protein metabolism, and relying on hormone signaling [3]. Plant hormones, especially abscisic acid (ABA), play pivotal roles in stress responses. For example, in response to drought stress, ABA levels increase rapidly, binding to receptors in the PYR1/PYL/RCAR (PYLs) family of proteins [4, 5]. The ABA-PYL complex then inhibits downstream protein phosphatases in clade A of the PP2C family [6], leading to the activation of sucrose non-fermenting-1-related protein kinase-2s. These kinases phosphorylate downstream effectors to initiate protective responses [7]. Under HM stress, the application of exogenous ABA has been shown to enhance plant tolerance by influencing various physiological, biochemical, and molecular processes [8]. To improve resistance to HM stress, several genes related to ABA signaling have been identified, including HM ATPases, ABC transporters, Zn iron permeases, natural resistance-associated macrophage proteins, and metallothioneins (MTs) [9, 10].

MTs are a superfamily of small (< 10 kDa) cytosolic cysteine (Cys)-rich metal ion-coordinating proteins [9, 11]. Plant MTs are categorized into four types (MT1, MT2, MT3, and MT4), differentiated by the arrangement of their Cys residues. MT1 features six Cys residues clustered at both the amino (N)- and carboxyl-terminal (C) regions, forming Cys-X-Cys motifs (X represents any amino acid other than Cys). The N- and C-termini of MT2 contain eight and six Cys residues, respectively, while MT3 includes four Cys residues at the N-terminal and six at the C-terminal. MT4 is distinct in having three Cys-rich domains, each with five or six conserved Cys residues, separated by 10 to 15 amino acids [11, 12]. Research has increasingly shown that MTs participate in numerous vital biological processes, including protection, resistance to metal toxicity, stress response control, and physiological homeostasis regulation [12, 13]. MTs facilitate the storage, transport, and homeostasis of essential metal ions, such as Zn^II^ and Cu^I^, and play a critical role in the detoxification of toxic metal ions, such as Cd^II^, Hg^II^, and Pb^II^ [13–16]. Their exceptionally high Cys content and thiolate residues offer additional protection against reactive oxygen species (ROS), leading to the formation of disulfide bridges and subsequent metal ion release [13–16]. In cassava, 10 *MT* genes have been found to exhibit tissue-specific expression, acting as ROS scavengers [17]. In maize, nine *MT* genes display specific expression patterns across 23 tissues at various developmental stages and show varied transcriptional responses to HM stresses such as Cu, Cd, and Pb [16]. Furthermore, *MT*s are increasingly recognized for their roles in abiotic stress tolerance, including drought, salt, and abnormal temperature changes [13, 17, 18]. These findings underscore the need for further detailed studies to elucidate the critical roles of plant *MT*s.

Understanding the complexities of metal chelates in MTs is crucial for developing effective strategies to manage metal contamination. MTs chelate metals through the sulfhydryl groups of Cys residues, forming coordinate covalent bonds [13–18]. The chelating capacity of MTs is influenced by the number and arrangement of Cys residues within their domains [17, 18]. Despite this, the function of the highly heterogeneous Cys-rich domain in plant MTs remains largely unexplored [19], and current evidence does not indicate that the N- or C-terminal Cys residues in *B. papyrifera* MTs impact their HM tolerance. Therefore, further research is required to investigate the role of Cys distribution in *B. papyrifera* MT proteins under HM stress.

To enhance our understanding of *B. papyrifera’s* adaptation mechanisms to HM-polluted environments and to support its efficient cultivation and utilization, *MT* genes were identified from transcriptomes based on their basic sequence characteristics, conserved domains, protein motifs, and phylogenetic relationships. Given the potential link between *MT*s and ABA signaling, four HMs—Cd, Cu, Mn, and Zn—along with ABA treatments, were employed to examine the expression of selected *MT* genes. A yeast expression system was utilized to explore the capability and mechanism of HM chelation by MTs. This study provides new insights that will inform future research on the role of *BpMTs* in HM remediation.

## Results

### Sequence characteristics and phylogenesis of *BpMT*s

Fifteen putative *BpMT* genes were initially identified from the *B. papyrifera* transcriptome. Following a conserved domain analysis and a comparison of homologous proteins using BLASTP, three genes lacking MT domains were excluded. Consequently, 12 genes were confirmed as *MT* family members. The open reading frames (ORFs) of these 12 *BpMT* genes ranged from 186 to 372 bp; their deduced peptides comprised 61 to 123 amino acids, and their molecular weights (MWs) varied from 15,473.77 to 29,546.96 Da. The theoretical isoelectric points (pI) of these MTs ranged from 5.24 to 5.32, and they were predicted to be localized in the nucleus, cytoplasm, cell membrane, and chloroplasts (Table 1).

**Fig. 1:**
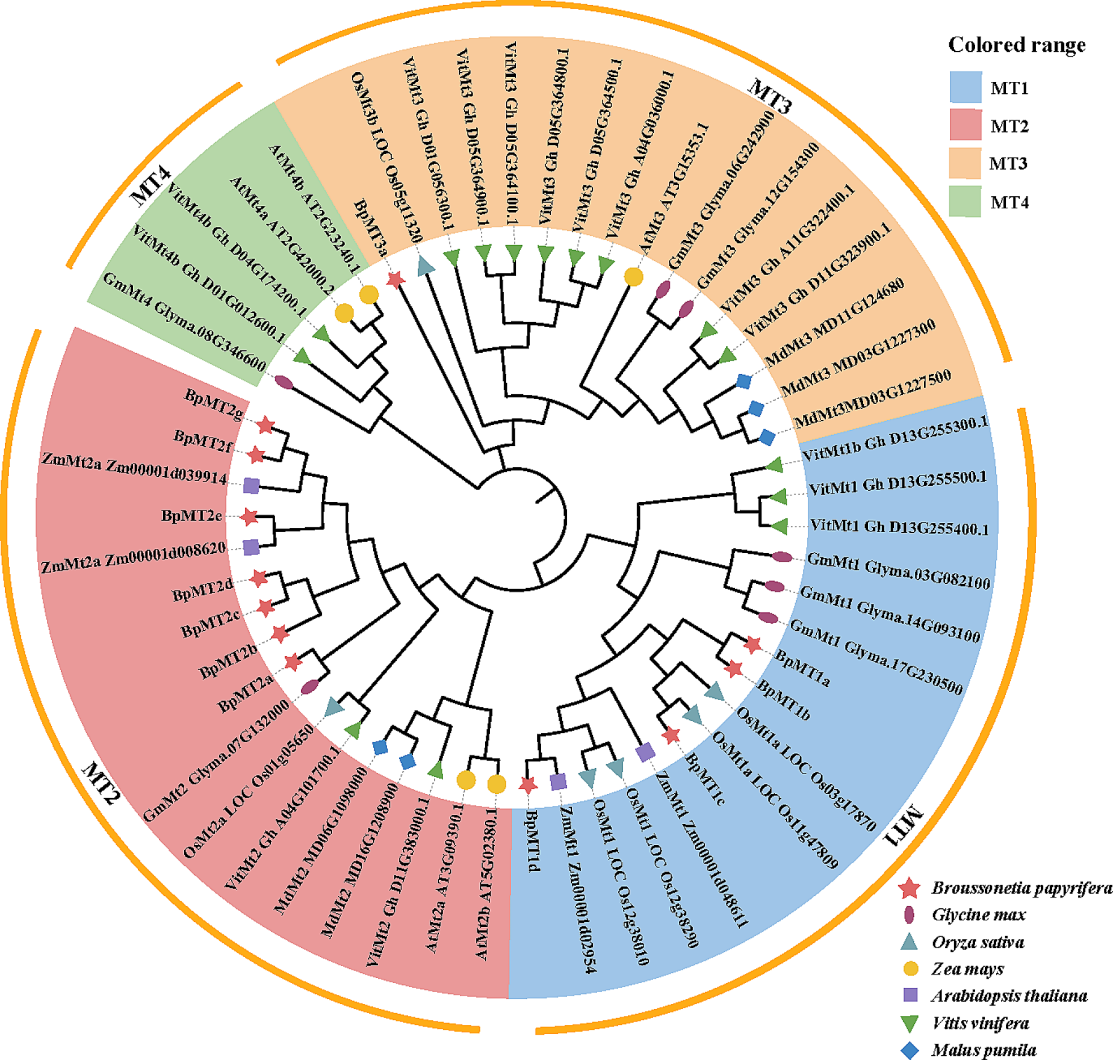
Phylogenetic relationship of BpMT proteins. Alignments of the MT proteins from *B. papyrifera* and other 6 species were performed with Clustal W. The phylogenetic tree was constructed using Neighbor-Joining method of MEGA7. Different colors mean different type members according to sequence similarity

To elucidate the phylogenetic relationships of BpMT proteins among *B. papyrifera* and other plant species, an unrooted phylogenetic tree was constructed. This tree was based on the alignment of MT protein sequences from *B. papyrifera*, *Glycine max*, *Oryza sativa*, *Zea mays*, *Arabidopsis thaliana*, *Vitis vinifera*, and *Malus pumila* using the neighbor-joining method. The evolutionary tree’s topology grouped all MT proteins into four subclasses, with the 12 identified BpMTs distributed among three types, excluding MT4. The MT2 type was the most populated with BpMTs, including BpMT2a, BpMT2b, BpMT2c, BpMT2d, BpMT2e, BpMT2f, and BpMT2g, whereas the MT3 type contained only BpMT3a. The remaining four genes, *BpMT1a*, *BpMT1b*, *BpMT1c*, and *BpMT1d*, fell under the MT1 type (Fig. 1).

**Table 1:** Information of the *MT* gene family in *B. papyrifera*

Gene names	Transcriptome No.	ORF/bp	Number of amino acids	Molecular weight/Da	Theoretical pI	Predicted locations
BpMT1a	TRINITY_DN232562_c1_g5	225	74	18333.82	5.29	Chloroplast/ Cytoplasm/ Nucleus
BpMT1b	TRINITY_DN232562_c1_g4	213	70	17356.82	5.29	Cell membrane/ Chloroplast/ Cytoplasm/ Nucleus
BpMT1c	TRINITY_DN232804_c0_g1	225	74	18119.51	5.30	Chloroplast/ Cytoplasm/ Nucleus
BpMT1d	TRINITY_DN232562_c1_g2	228	75	18324.97	5.28	Cytoplasm/ Nucleus
BpMT2a	TRINITY_DN235290_c0_g1	237	78	19096.65	5.28	Cytoplasm
BpMT2b	TRINITY_DN234165_c0_g2	300	99	24090.86	5.29	Nucleus
BpMT2c	TRINITY_DN232562_c2_g1	243	80	19336.53	5.31	Cell membrane/ Cytoplasm/Nucleus
BpMT2d	TRINITY_DN235814_c2_g1	372	123	29546.96	5.24	Cell membrane
BpMT2e	TRINITY_DN235814_c1_g1	249	82	20096.02	5.26	Cell membrane/Cytoplasm
BpMT2f	TRINITY_DN202585_c0_g1	237	78	19072.28	5.32	Cytoplasm
BpMT2g	TRINITY_DN235814_c0_g1	237	78	19240.51	5.32	Cytoplasm
BpMT3a	TRINITY_DN210650_c0_g1	186	61	15473.77	5.30	Cytoplasm

**Fig. 2:**
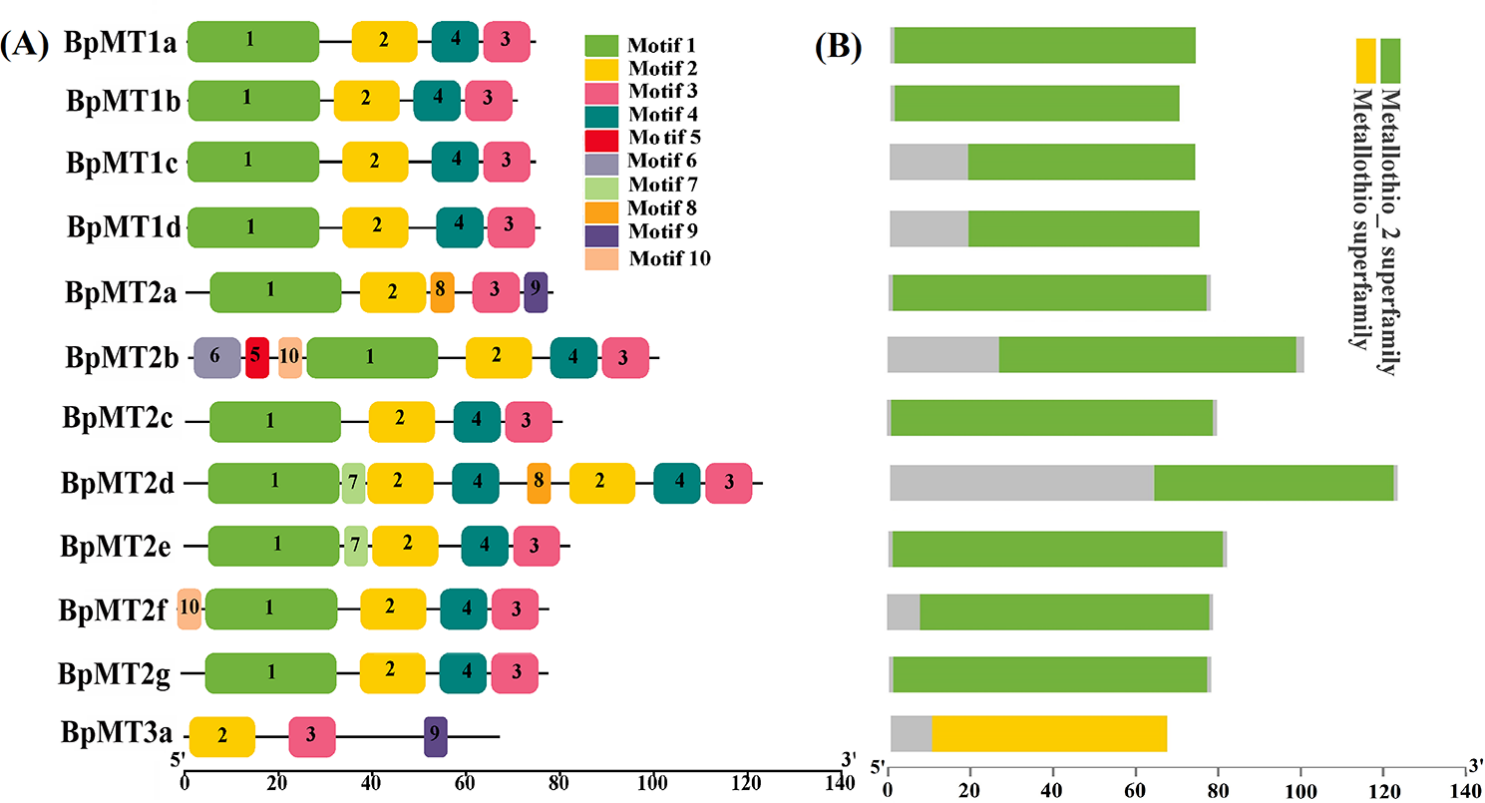
The conserved motifs and domains of BpMT proteins. (**A**) The motifs distributed in the 12 BpMT proteins, the 10 conserved motifs are represented with different color boxes. The dark line shows the length of proteins. (**B**) Distribution of conserved domains within BpMT proteins. The relative positions of each domain are shown in color boxes, the names are indicated on the right

**Fig. 3:**
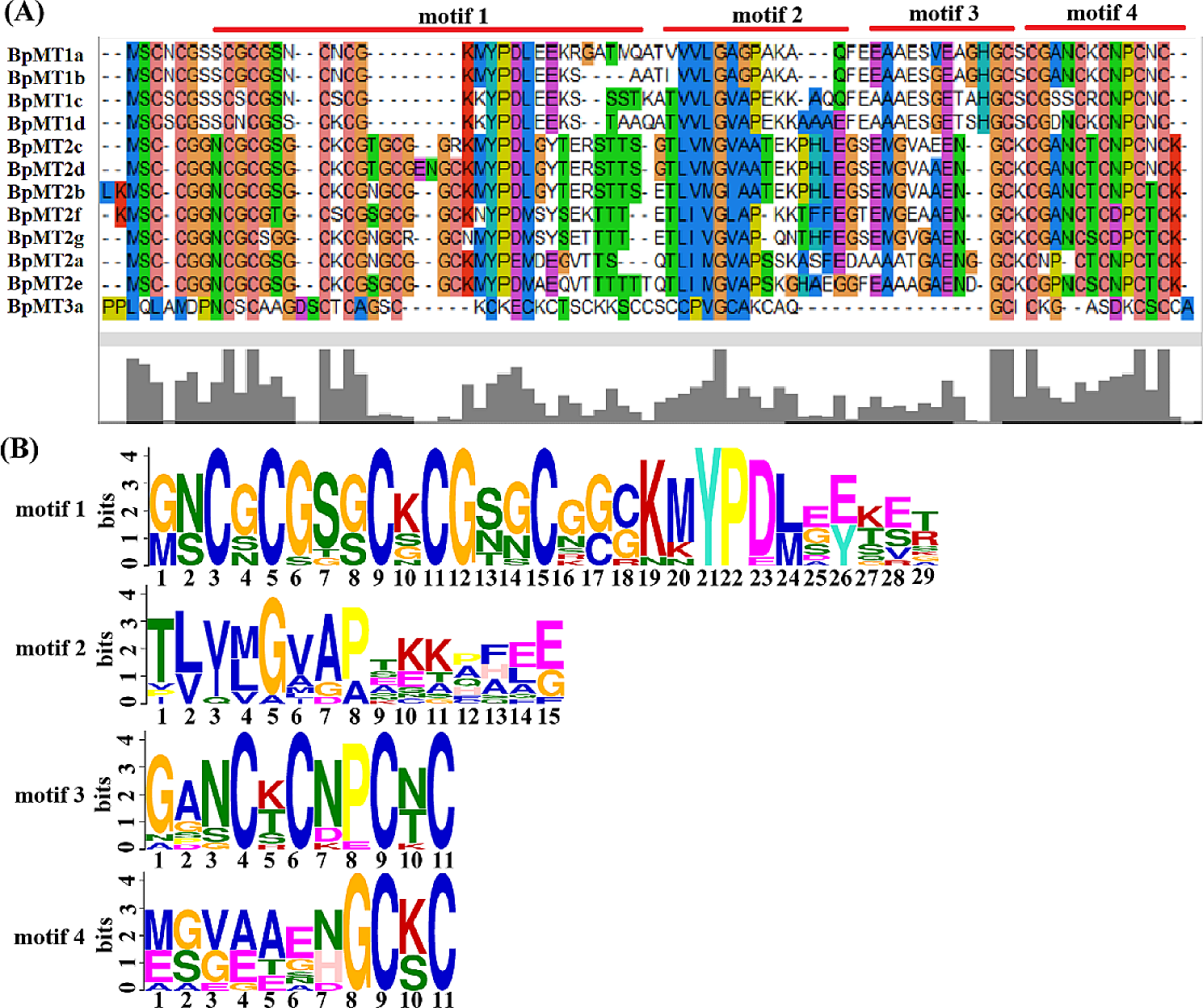
Multiple sequence alignment of the motifs of 12 BpMT proteins (**A**) and the sequence logo of motif1, motif2, motif3 and motif4 (**B**)

The motif composition analysis further validated the subclassification and revealed the conserved domain characteristics of these proteins. Using the MEME tool [24], 10 conserved motifs, each comprising 6–29 amino acids, were identified across the 12 BpMT proteins (Table 2). The most prevalent motifs included motif1, motif2, motif3, and motif4 (Fig. 2A). Motifs 2 and 3 were present in all BpMT proteins, motif1 was found in 11 of the BpMTs except for BpMT3a, and motif4 appeared in 10 of the proteins, excluding BpMT3a and BpMT2a. The amino acid sequences of motifs 1, 2, 3, and 4 were highly conserved, corresponding to the metallothio domain (Fig. 3). Among the BpMTs, BpMT2d possessed the most motifs (a total of eight). BpMT2a, BpMT2e, and BpMT2f each had five motifs, sharing motif1, motif2, and motif3. The unique motifs 10 and 4 in BpMT2f differentiated it from BpMT2a, which uniquely features motifs 8 and 9. Motif7 was exclusive to BpMT2e. BpMT3a had the fewest motifs, with only three—motif2, motif3, and motif9. The other six BpMTs shared four motifs: motif1, motif2, motif4, and motif3 (Fig. 2A). Additionally, PFAM analysis indicated that 11 of the BpMT proteins contained a unique metallothio_2 superfamily domain, while BpMT3a was characterized by a single metallothio superfamily domain (Fig. 2B). The metallothio_2 domain, richer in Cys residues than the metallothio domain, aligns closely with Class II MTs, suggesting a distinct specificity for BpMT3a compared to the other 11 BpMTs, warranting further confirmation and functional characterization of BpMT3a.

**Table 2:** Motif sequences of BpMT proteins identified by MEME tool

Motif	Width	Best possible match
Motif 1	29	GNCGCGSGCKCGSGCGGCKMYPDLEEKET
Motif 2	15	TLVLGVAPTKKPFEE
Motif 3	11	GANCKCNPCNC
Motif 4	11	EGVAAENGCKC
Motif 5	6	KIFDDL
Motif 6	11	MGVQDQEEKKV
Motif 7	6	TTSGTQ
Motif 8	6	DAERAT
Motif 9	6	NGCICK
Motif 10	6	KMSCCG

### Expression patterns of *BpMTs* in response to HM stresses

**Fig. 4:**
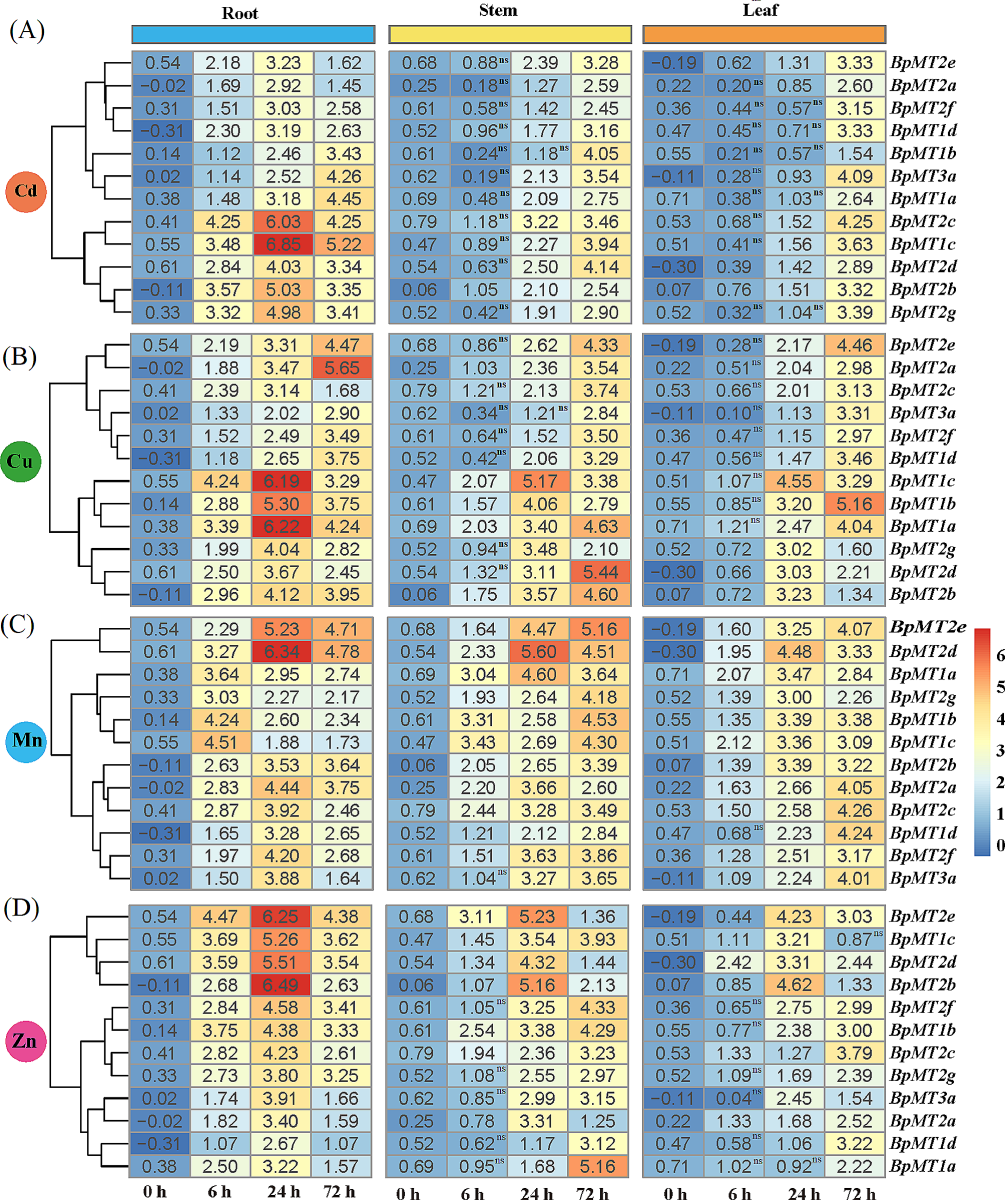
Expression patterns of *BpMT* genes under different HM stresses for 0, 6, 24, 72 h in the roots, stems and leaves. Heatmap of *BpMT* genes expression data was created by Tbtools with row clustering applied, and the blue/yellow/orange colors indicated the low/medium/high expression level. The expression was related to the internal reference gene and control conditions. (**A**) Cd stress. (**B**) Cu stress. (**C**) Mn stress. (**D**) Zn stress. The data followed by ‘ns’ means the difference between the time point and 0 h was not significant in the same tissue, while the data not marked were all significantly different to 0 h (*P* < 0.05)

To assess the potential function of *BpMTs* in HM stress responses, the expression patterns of 12 *BpMT*s were evaluated under stresses from CdCl_2_ (Cd), CuSO_4_ (Cu), MnCl_2_ (Mn), and ZnCl_2_ (Zn). Interestingly, all *BpMT*s showed high induction and reached peak expression levels in the roots, while generally exhibited the lowest and least induction in the leaves (Fig. 4).

#### Under Cd stress

The 12 *BpMTs* exhibited diverse expression profiles with tissue-specific responses, clustering into three groups. Group one, including *BpMT2e*, *BpMT2a*, *BpMT2f*, and *BpMT1d*, showed relatively lower expression levels compared to the other groups, peaking at 24 h in the roots. Another group comprised *BpMT1b*, *BpMT3a*, and *BpMT1a*, displaying intermediate transcription levels. The most significantly induced group included *BpMT2c*, *BpMT1c*, *BpMT2d*, *BpMT2b*, and *BpMT2g*. Notably, *BpMT2c* and *BpMT1c* were highly upregulated by Cd stress; in the roots, *BpMT2c’s* expression at 6 h ranged from 1.19- to 3.79-fold higher than the other *BpMTs*, and at 24 and 72 h, *BpMT1c’s* expression was 1.14- to 2.78- and 1.17- to 3.6-fold higher, respectively. In the leaves, *BpMT2c’s* expression increased 1.04- to 2.75-fold compared to other *BpMTs* (Fig. 4A). Furthermore, *BpMT2g* showed a high correlation with the other seven *BpMT*s under Cd stress (Fig. S1A).

#### Under Cu stress

The response levels of the 12 *BpMTs* to Cu stress were generally higher than under Cd stress, with expression values frequently exceeding 5.0 and 4.0. The *BpMTs* were grouped into two classes based on their transcription patterns. Class one included *BpMT1c*, *BpMT1b*, *BpMT1a*, *BpMT2g*, *BpMT2d*, and *BpMT2b*, whose transcription activities were slightly more pronounced than those of the second class comprising the remaining six *BpMTs*. In the roots, peak expressions for class one and *BpMT2c* occurred at 24 h, with *BpMT1a*, *BpMT1c*, and *BpMT1b* showing the highest expressions at 1.51-~3.09-fold, 1.50-~3.06-fold, and 1.29-~2.63-fold higher than the other nine *BpMTs*, respectively. In the stems, except for *BpMT1c*, *BpMT1b*, and *BpMT2g* peaking at 24 h with values of 5.17, 4.06, and 3.48, respectively, the other nine genes reached their maximum transcription at 72 h. In the leaves, eight *BpMTs*, excluding *BpMT1c*, *BpMT2g*, *BpMT2d*, and *BpMT2b*, were most expressed at 72 h, with *BpMT1b* recording the highest transcription at 3.85-fold greater than *BpMT2b* (Fig. 4B). Additionally, *BpMT2d* and *BpMY3a* showed significant correlations with the other four *BpMT*s (Fig. S1B), indicating that most *BpMTs* might be crucial genes collectively responding to Cu stress.

#### Under Mn stress

All 12 *BpMT* genes exhibited more pronounced induction than under Cd and Cu stresses and were divided into three groups based on their expression patterns. Group 1, displaying the most significant expression, included *BpMT2e* and *BpMT2d*. Group 2 consisted of *BpMT1a*, *BpMT2g*, *BpMT1b*, and *BpMT1c*. The remaining six genes (*BpMT2b*, *BpMT2a*, *BpMT2c*, *BpMT1d*, *BpMT2f*, and *BpMT3a*), forming Group 3, reached peak expression at 24 h, except for *BpMT2b*. In the roots, *BpMT2d’s* expression was 3.37 times that of *BpMT1c*. In the stems, *BpMT2d* showed the highest expression at 24 h, with transcription levels 1.22 to 2.64 times those of other *BpMTs*. In the leaves, half of the *BpMTs* had expression values exceeding 4.00 (Fig. 4C). Correlation analysis revealed that *BpMT1d* and *BpMT2a* were significantly associated with *BpMT2b*, *BpMT2d*, *BpMT2e*, and *BpMT2f*, suggesting a synergistic response to Mn stress (Fig. S1C).

#### Exposure to Zn stress

All *BpMTs* genes were upregulated and categorized into three groups. Group 1 consisted of *BpMT2e*, *BpMT1c*, *BpMT2d*, and *BpMT2b*. Group 2 included *BpMT2f*, *BpMT1b*, *BpMT2c*, and *BpMT2g*, while Group 3 comprised *BpMT3a*, *BpMT2a*, *BpMT1d*, and *BpMT1a*. In the roots, all *BpMTs* were significantly induced, showing maximum expression at 24 h, although *BpMT1d’s* expression was only 41% of *BpMT2b’s*. In the stems, *BpMT2e* and *BpMT1b* were notably upregulated at 6 h, while by 72 h, *BpMT1a* was the most highly transcribed, reaching levels 1.19 to 4.13 times those of other *BpMTs*. In the leaves, *BpMT2b* and *BpMT2e* were most prominent, with expression values 1.40 to 5.02 times and 1.28 to 4.60 times that of the other 10 *BpMTs*, respectively (Fig. 4D). Furthermore, all *BpMT*s exhibited significant co-expression with three other *BpMT*s (Fig. S1D), highlighting a broad co-expression network within the *BpMT* family in response to Zn stress, particularly involving *BpMT2c* and *BpMT2d*.

### Transcription potential of *BpMTs* mediated by ABA signaling

**Fig. 5:**
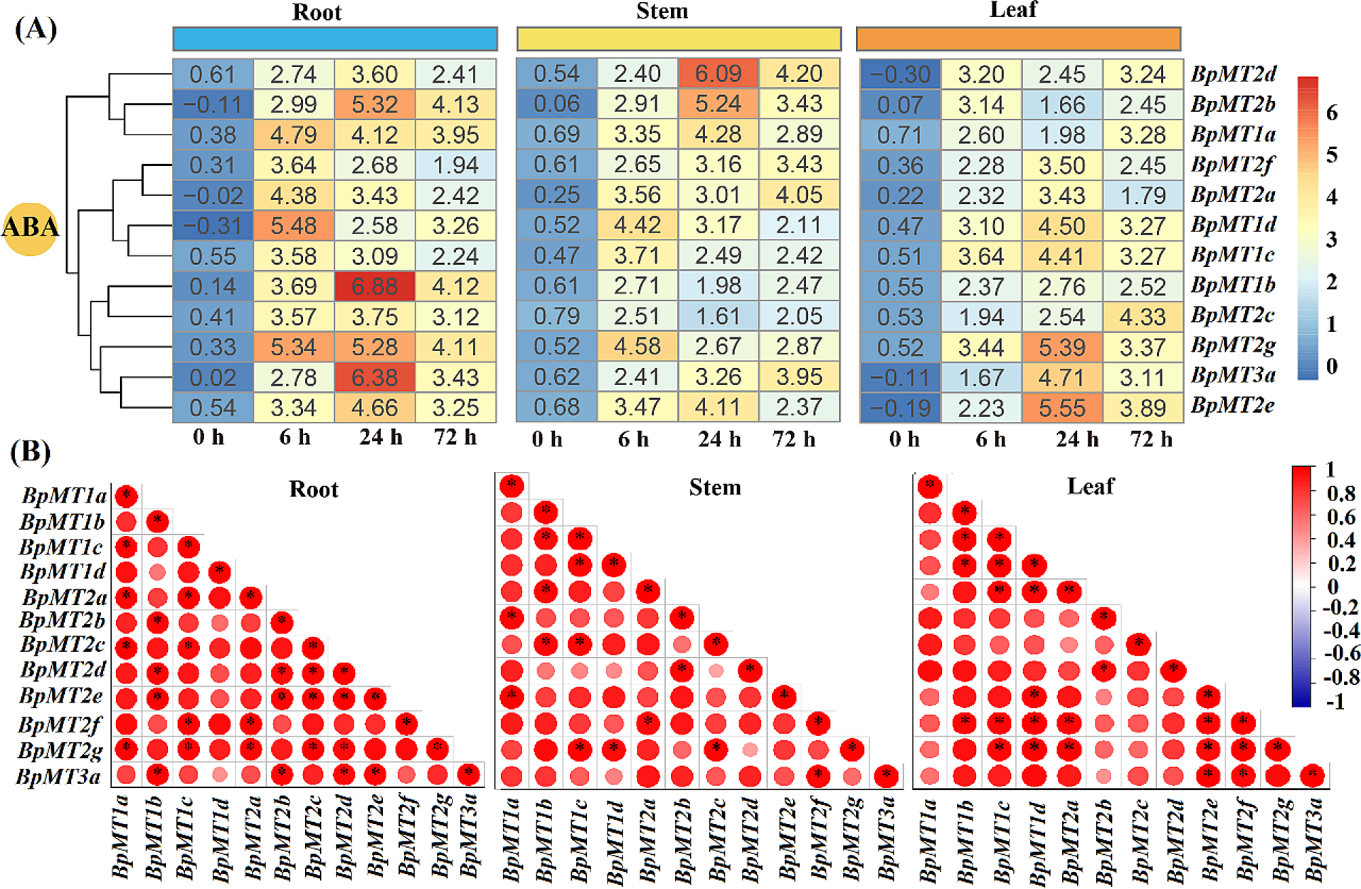
Expression profiles and relativities of *BpMT* genes under ABA treatment for 0, 6, 24, 72 h in the roots, stems and leaves. The expression is relative to the internal reference gene and at control conditions. The heatmap of expression data was created by Tbtools same as Fig. 4. All the differences of each gene between different time points and 0 h were significant in the same tissue (*P* < 0.05). (**A**), the expression files. (**B**), the relativities

To explore whether *BpMT*s participate in ABA signaling, the transcriptional responses of 12 *BpMT*s to ABA treatment were examined. The results demonstrated significant upregulation of all *BpMT* genes by ABA across roots, stems, and leaves during differentiation (Fig. 5A). In the roots, half of the 12 *BpMTs* (*BpMT1a*, *BpMT2f*, *BpMT2a*, *BpMT1d*, *BpMT1c*, and *BpMT2g*) reached their maximum expression levels at 6 h, all exceeding a value of 3.5. The remaining six *BpMTs* peaked at 24 h with expression values ranging from 3.60 to 6.88; notably, *BpMT2f* and *BpMT3a* were the most prominent, with values of 6.88 and 6.38, respectively. In the stems, the highest expression values at 6, 24, and 72 h involved different subsets of five, four, and three *BpMTs*, respectively. *BpMT2d* (6.09) exhibited the most significant induction at all measured time points, ranging from 1.16 to 3.78 times that of other genes. In the leaves, while *BpMT2d*, *BpMT2b*, and *BpMT1a* experienced a trough in expression at 24 h, *BpMT2f* increased to its peak at 24 h. The remaining eight *BpMT* genes reached maximum transcription at 24 h (Fig. 5A), suggesting a complex relationship between *BpMTs* and ABA signaling.

**Fig. 6:**
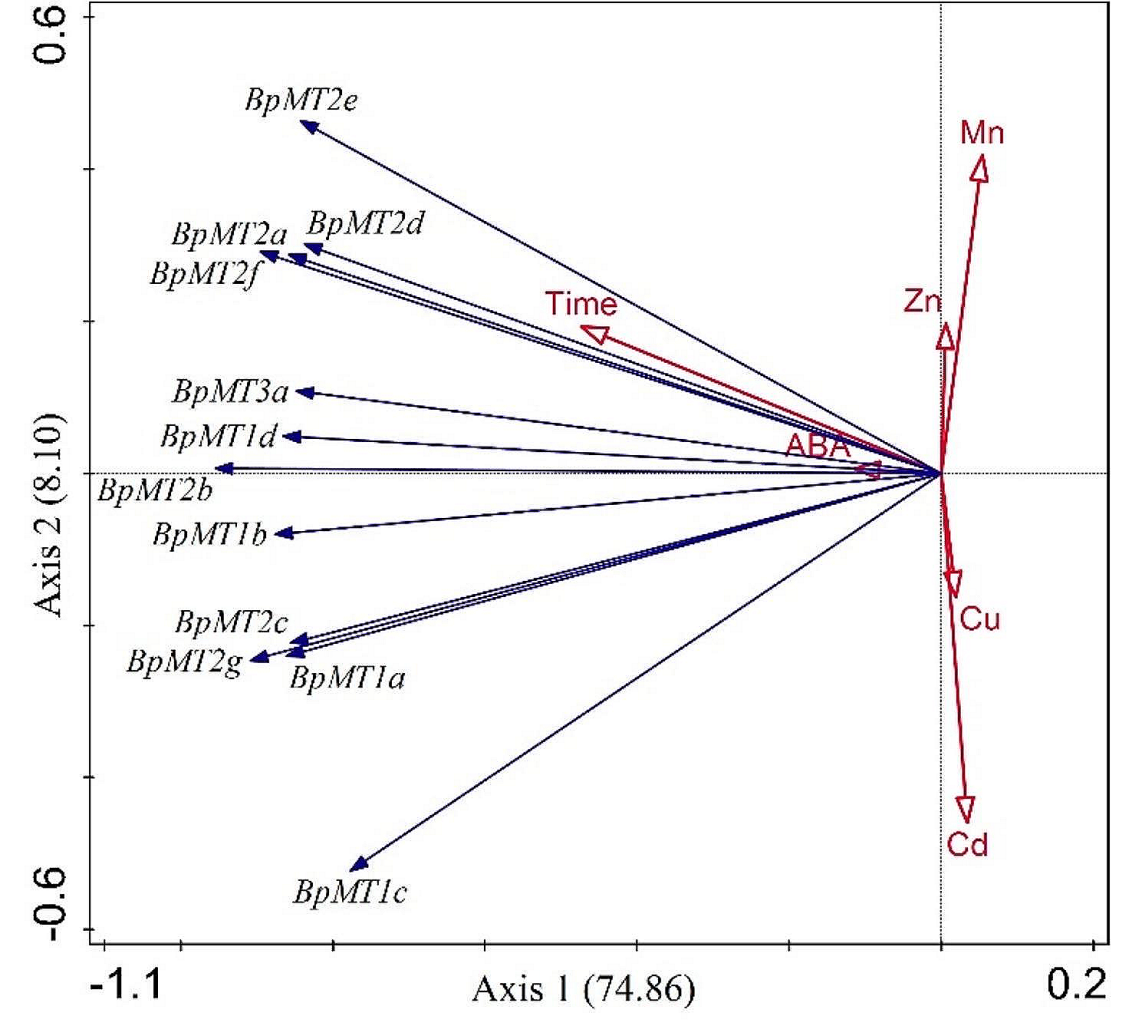
Redundancy analysis (RDA) was employed using Canoco 5.0 for gene expression and experiment treatments, including heavy metal and ABA treatments at different time points (0, 6, 24, 72 h)

To determine if there was a functional relationship mediated by ABA signaling among these genes, correlation analysis was performed on the expression levels post-ABA treatment. In the roots, except for *BpMT1d*, all other *BpMT*s showed significant correlations with at least two other *BpMT*s. In stems, every *BpMT* gene exhibited significant correlations with another *BpMT*. Conversely, in the leaves, *BpMT1a* and *BpMT2c* did not correlate with any other *BpMT*s (Fig. 5B). These findings suggest that *BpMT*s response widely and deeply to ABA compared to HM stress (Fig. 6), indicating a potential reliance of *BpMT*s on the ABA signaling pathway.

### Heterologous expression of *BpMTs* conferring HM tolerance in yeast

**Fig. 7:**
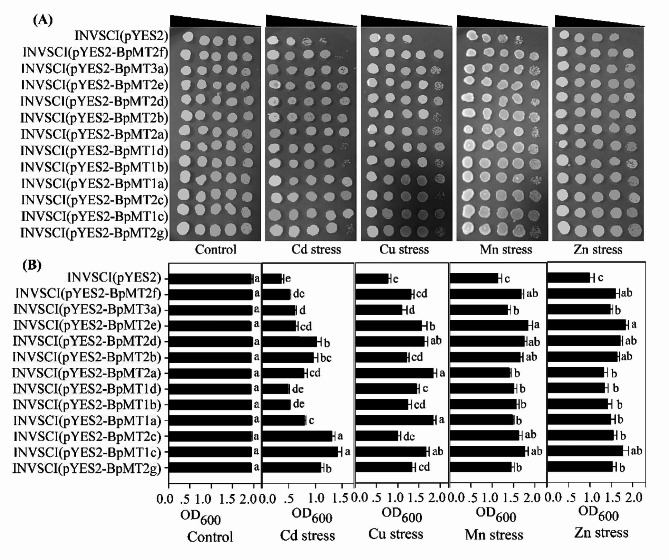
Analysis of the heavy metal stress response function of *BpMT* genes in yeast. (**A**) The growth performance of the control and *BpMT*s transformed yeasts under HM stresses. The decreasing trend triangle above represents the dilution ratio of 0^0^, 10^− 1^, 10^− 2^, 10^− 3^, 10^− 4^. (**B**) The yeast cell densities (OD_600_) of INVSCI (pYES2) and INVSCI (pYES2-BpMTs) under four HM treatments. Control was cultivated in a normal media without HM stress. The lowercase letters in the figure indicated the significant differences (*P* < 0.05) among different yeast lines

To investigate the role of *BpMT* genes in response to HM stimuli, all 12 *BpMT*s were individually expressed in yeast for HM stress resistance analysis. The yeast strain INVSCI harboring pYES2-BpMTs or control vector pYES2 was exposed to Cd, Cu, Mn, and Zn stress. Yeast clones were diluted 10^0^-, 10^1^-, 10^2^-, 10^3^-, and 10^4^-fold, respectively, and spotted onto Sc-Ura plates. Under normal conditions, the growth and viability of INVSCI (pYES2) and INVSCI (pYES2-BpMTs) cells were comparable. However, under HM stress, INVSCI (pYES2) exhibited reduced or weaker growth compared to INVSCI (pYES2-BpMTs), particularly under Cu stress and at the highest dilution of 10^4^ (Fig. 7A). Optical density (OD_600_) values of INVSCI (pYES2) and INVSCI (pYES2-BpMTs) were recorded under Cd, Cu, Mn, and Zn stress. While there were no significant differences under normal conditions, notable discrepancies were observed after exposure to HM stress. INVSCI (pYES2-BpMTs) consistently demonstrated higher OD_600_ values than INVSCI (pYES2), particularly under the HM conditions. Apart from INVSCI (pYES2-BpMT2f), INVSCI (pYES2-BpMT1d), and INVSCI (pYES2-BpMT1b) under Cd stress, and INVSCI (pYES2-BpMT2c) under Cu stress, all other yeast strains subjected to the four HM stresses exhibited significantly greater growth activities than the control INVSCI (pYES2) (Fig. 7B). These findings suggest a beneficial role of *BpMTs* in mediating HM stress responses.

### *BpMT-*transformed yeast cells displayed higher HM accumulations

**Fig. 8:**
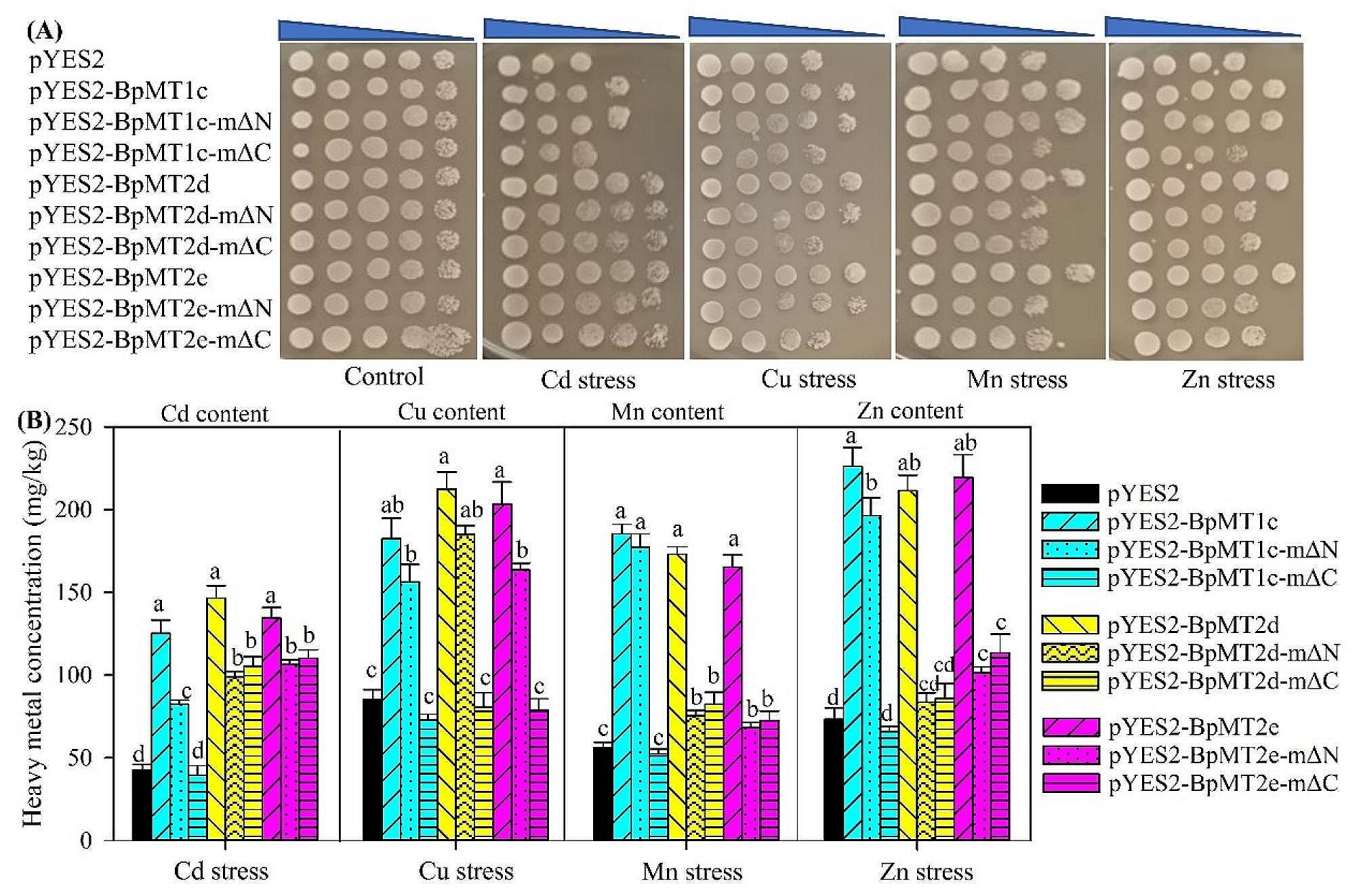
HMs tolerance and accumulation study of yeast *BpMT*s’ mutants (N- and C-domain named as mΔN and mΔC, respectively) generated by site-directed mutagenesis in yeast. (**A**), Serially diluted transformed yeast INVSC1containing pYES2, pYES2-BpMT1c, pYES2-BpMT1c-mΔC, pYES2-BpMT1c-mΔN, pYES2-BpMT2d, pYES2-BpMT2d-mΔN, pYES2-BpMT2d-mΔC, pYES2-BpMT2e, pYES2-BpMT2e-mΔN, pYES2-BpMT2e-mΔC spotted on Sc-U selective media with and without metal (Control), Cd, Cu, Mn, Zn. (**B**), HMs accumulation in yeast cells according to (A). All data are mean ± SD (*n* = 3). Different letters above bars indicate significant differences among different yeast strains under the same HM stress (*P* < 0.05)

To assess the link between HM stress tolerance and metal accumulation, ICP-MS analysis was conducted to measure the concentrations of Cd, Cu, Mn, and Zn in yeasts transformed with *BpMT1c*, *BpMT2d*, and *BpMT2e*. Results depicted in Fig. 8 show that, compared to the control yeast, yeast cells expressing *BpMT1c*, *BpMT2d*, and *BpMT2e* accumulated significantly higher levels of Cd (2.94-, 3.45-, and 3.16-fold increase), Cu (2.14-, 2.49-, and 2.38-fold increase), Mn (3.29-, 3.07-, and 2.93-fold increase), and Zn (3.09-, 2.89-, and 3.00-fold increase) respectively, when supplemented with 20 µM Cd, 50 µM Cu, 20 µM Mn, and 10 µM Zn. However, between the control yeast and yeast strains transformed with *BpMT1c*, *BpMT2d*, and *BpMT2e*, no significant differences in growth were observed under non-stressed conditions (Fig. 8A). This indicates that overexpression of *BpMTs* enhances yeast resistance to HMs through increased metal accumulation.

### Type 1 and 2 *BpMTs* depend differently on N- and C-terminal Cys-rich domains in HM toxicity resistance

To elucidate the mechanisms of *BpMT*s’ tolerance and chelation capabilities toward HMs, N- and C-terminal Cys residues were independently mutated and cloned into pYES2, generating mutated yeast expression recombinants of *BpMT1c*, *BpMT2d*, and *BpMT2e*. INVSC1 yeast strains harboring pYES2, pYES2-BpMT1c, pYES2-BpMT1c-mΔN, pYES2-BpMT1c-mΔC, pYES2-BpMT2d, pYES2-BpMT2d-mΔN, pYES2-BpMT2d-mΔC, pYES2-BpMT2e, pYES2-BpMT2e-mΔN, and pYES2-BpMT2e-mΔC were subjected to spot and growth assays under various HM stresses. The spot assay results indicated that all mutants exhibited reduced growth compared to their non-mutant counterparts (pYES2-BpMT1c, pYES2-BpMT2d, and pYES2-BpMT2e). Specifically, INVSC1(pYES2-BpMT1c-mΔC) displayed more growth inhibition than INVSC1(pYES2-BpMT1c-mΔN). Conversely, INVSC1(pYES2-BpMT2d-mΔC) and INVSC1(pYES2-BpMT2e-mΔC) did not show obvious inhibition compared to INVSC1(pYES2-BpMT2d-mΔN) and INVSC1(pYES2-BpMT2e-mΔN) respectively under Cd, Mn, and Zn stresses (Fig. 8A). No significant growth differences were observed under control conditions (Fig. 8A). ICP-MS analysis to quantify HM accumulation revealed that INVSC1(pYES2-BpMT1c-mΔC) accumulated only 31.57%, 40.12%, 28.21%, and 29.12% of the Cd, Cu, Mn, and Zn levels, respectively, seen in INVSC1(pYES2-BpMT1c). INVSC1(pYES2-BpMT2d-mΔC) and INVSC1(pYES2-BpMT2e-mΔC) chelated slightly more HMs than INVSC1(pYES2-BpMT2d-mΔN) and INVSC1(pYES2-BpMT2e-mΔN); however, most differences were not statistically significant excluding under Cu stress (Fig. 8B). The HM accumulation in INVSC1(pYES2-BpMT2d-mΔN) and INVSC1(pYES2-BpMT2e-mΔN) was 67.51% and 79.18% (Cd), 87.15% and 80.38% (Cu), 43.52% and 41.27% (Mn), 39.49% and 46.11% (Zn) of INVSC1(pYES2-BpMT2d) and INVSC1(pYES2-BpMT2e), respectively (Fig. 8B). These findings demonstrate the significant roles of *BpMT1c*, *BpMT2d*, and *BpMT2e* in HM detoxification, which is mediated through Cys residues located in their C-terminal or N-terminal domains.

## Discussions

Plant *MT* genes are crucial components that respond to various environmental stimuli [12–18]. To inform scientific strategies for the cultivation and management of paper mulberry, understanding the molecular mechanisms of adaptation to adverse conditions is imperative. In this context, the transcriptomes of paper mulberry plants under different HM stresses were sequenced to establish a foundational database for identifying key genes that adapt or respond to environmental stresses. Owing to their significant transcription, abundant presence, and crucial roles in plant metal homeostasis and detoxification [9, 11], 12 *BpMT* genes were identified from the aforementioned transcriptome data. Based on their evolutionary relationships, these 12 *BpMT*s are classified into three types of MTs (MT1–3), excluding type MT4 (Fig. 1), which also influenced the nomenclature of *BpMT*s. Plant *MT*s are generally categorized into four types (MT1 ~ 4) [14, 19, 20]. However, in this study, the identified 12 *BpMT* genes did not include type *MT4*, likely because the *BpMT* family genes examined are shared genes screened from the root, stem, and leaf transcriptomes under various stresses, primarily representing transcriptionally expressed genes responsive to HM stress. This screening does not ensure that all members are induced under HM stress, nor does it allow for the identification of other tissue-specific genes. Type 4 MTs are typically confined to developing and mature seeds and diminish post-germination [12, 21]; hence, the 12 *BpMT*s identified from the root and leaf transcriptomes did not encompass any type 4 *BpMT*s. Nonetheless, the screening of these genes was predicated on their transcriptional expression under stress, which may relate to the regulation of HM stress.

To understand the biological functions of *BpMT*s, their evolutionary relationships were analyzed initially, revealing that the 12 BpMT proteins shared high similarity with MTs from other species such as *A. thaliana*, *O. sativa*, and *V. vinifera* (Fig. 1). Based on conserved domain analysis, 11 BpMTs were classified within the ‘metallothio_2 superfamily’ domain, whereas BpMT3a was associated with the ‘metallothio superfamily’ (Fig. 2B). Additionally, the 12 BpMTs exhibited various motif compositions, with the main motifs presumed to be markers of the metallothio superfamily [9, 22]. This foundational information confirmed the classification of the 12 *BpMT*s within the MT protein family.

To determine the involvement of these *BpMTs* in the HM stress response, the expression activities of the 12 *BpMTs* were analyzed under treatments with Cd, Cu, Mn, and Zn in roots, stems, and leaves. It was observed that all 12 *BpMTs* were inducible by these HMs, displaying tissue-specific expression patterns, particularly *BpMT2c* and *BpMT1c* in response to Cd, *BpMT1a* and *BpMT1c* to Cu, *BpMT2d* to Mn, and *BpMT2e* and *BpMT2b* to Zn (Fig. 4). The transcriptional activity of genes in response to stress can effectively predict their potential functions. For instance, nine *MT* genes in maize were induced with diverse expression patterns in response to Cu, Cd, and Pb stress, suggesting their role in maize’s resistance to HMs [16]. *SaMT3* from *Sedum alfredii*, upregulated by Cd stress, was further confirmed to promote plant Cd tolerance by scavenging [22]. The chickpea *MT1* gene displayed inconsistent upregulation against major toxic HMs, such as As [As(III) and As(V)], Cr(VI), and Cd toxicity; further assays confirmed that *MT1* overexpression in transgenic lines could reduce As(V) and Cr(VI) accumulation, while enhance Cd accumulation; *Arabidopsis* transformed with the *MT1* gene showed mitigated HM stress [23]. *Canavalia rosea MT*s exhibit habitat- and environmental stress-regulated patterns, which likely contributed to thermotolerance [15]. These reports, along with the inducible expression of *BpMT*s, suggest that *BpMT* genes play roles in HM stress responses. From the expression levels and relationships observed, the responses to Cd and Cu, Zn and Mn were more correlated than to Zn and Mn, Cd and Cu, accordingly; *BpMT1c* was particularly notable in the response to Cd and Cu, while *BpMT2e* and *BpMT2d* were significant in the response to Mn and Zn stress (Figs. 4 and 6, S1).

To further confirm the function of *BpMTs* in response to HM stress, 12 *BpMTs* were individually inserted into pYES2 vectors and transformed into INVSCI yeast cells for expression assays. The results demonstrated that transgenic yeasts expressing *BpMTs* exhibited improved growth compared to control yeast under HM conditions (Cd, Cu, Mn, and Zn) (Fig. 7), underscoring the beneficial role of *BpMTs* in mitigating HM stress. The yeast expression system is commonly employed as a rapid and straightforward method for investigating the functions of target genes, as evidenced by studies involving *Caragana korshinskii CkMT4* [21], *Silene vulgaris SvMT3* [24], and *Leymus chinensis LcMT3* [25]. These findings further validate the effectiveness of *BpMTs* in the yeast expression system and their potential roles in regulating plant responses to HM stress.

The presence of Cys-rich domains is crucial for the chelation of metal ions [13–16]. Tolerance to HM stress or chelation is directly linked to the N- or C-terminal Cys-rich domains, although the specific roles of these domains can vary by species [17–21]. In this study, we observed that tolerance to Cd, Cu, Mn, and Zn stresses is not uniformly influenced by these domains across different types of *BpMT*s. The C-terminal domain exhibited a more pronounced response in type 1 *MT* genes than the N-terminal domain, whereas both C- and N-terminals played similar roles in type 2 *MT* genes in *B. papyrifera* (Fig. 8). This observation aligns with the function *OsMT-I-Id*, a type 1 *MT* from rice, which responds to Cd, Cu, and As stress [19], and underscores the characteristic features of type I *MT*s potentially conferred by the six C-X-C motifs distributed evenly across the N- and C-terminal domains [13–17]. In contrast, type 2 *MT*s are typically linked to HM detoxification [26]. Research by Singh et al. (2019) showed that both N- and C-terminal domains of *CjMT2* bind Cd^2+^ ions [27]. Additionally, Li et al. (2023) reported that mutations in the C-terminal Cys residues of *NtMT2F* significantly impaired its HM chelation capacity [28]. Our findings revealed that mutations in the N- and C-terminal Cys residues of *BpMT2d* and *BpMT2e* notably reduced HM chelation, with no significant differences observed between N- and C-terminal mutations (Fig. 8). This suggests that the N- and C-terminals of type 2 *MT*s may function similarly in HM tolerance and chelation, with the N-terminal Cys domain’s slightly superior performance potentially due to a higher count of Cys residues compared to the C-terminus. This hypothesis merits further investigation to pinpoint the specific Cys residues involved.

Additionally, the ABA-dependent signaling pathway is well-known for its central role in abiotic stress responses and involves numerous genes. Further investigation is required to determine whether the chelation of MT with HMs depends on ABA signaling. To examine whether the response of *BpMTs* to HM stress is associated with ABA, *B. papyrifera* was treated with ABA concurrently with HM treatments (0, 6, 24, and 72 h), and the transcription levels of each *BpMT* were analyzed. All 12 *BpMT*s were significantly induced by ABA to varying extents (Fig. 5A). Additionally, the expression profiles of some *BpMTs* in each tissue under ABA treatment mirrored those under HM stress (Figs. 4 and 5A), indicating that *BpMT* transcription is sensitive to ABA, similar to other reported ABA-related genes. For example, cucumber *MT* family genes (*CsMT2*, *CsMT3*, and *CsMT4*) can be induced by NaCl, PEG, and ABA and are thought to depend on ABA signaling [29]. mRNA levels of *CkMT4* are upregulated by ABA, and further studies have shown that *CkMT4* facilitates the re-establishment of desiccation tolerance in *C. korshinskii* seeds through ABA signaling [21]. Comparative expression analysis of *MT* genes from rice and *Arabidopsis* also supports the broad stress response of *MT*s to ABA [30]. Notably, the responses of all *BpMT*s to Cd, Cu, Mn, and Zn were significantly correlated with ABA, especially those of *BpMT1d*, *BpMT2b*, *BpMT1b*, and *BpMT3a* (Figs. 5B and 6). Therefore, based on the behavior of *BpMTs* under both HM stress and ABA treatment in this study, alongside previous findings, it is suggested that the response of *B. papyrifera MT* family members to HM stress is linked to ABA signaling.

Moreover, plants grow in soil, and their roots are the first to come into contact with HMs. An effective response from the root system is a crucial pathway through which plants adapt to and transport HMs. It is common for members within the same family to co-express or interact with each other to fulfill their functions; for example, in *Betula platyphylla*, BplMYB46 can form homodimers and heterodimers with BplMYB6, BplMYB8, BplMYB11, BplMYB12, and BplMYB13 [31]. In walnuts, JrWRKY2 and JrWRKY7 can form homodimers and interact with each other in response to abiotic stress [32]. In peaches, pERF98-1/2 forms homodimers/heterodimers and interacts with two PpERF1 proteins to amplify the jasmonate/ethylene signaling pathway [33]. However, reports on co-expression among *MT* family members are scarce. To elucidate the functional relationships within the *MT* family, we analyzed the expression correlation of the 12 *BpMT*s in the roots under various HM stresses and found significant correlations among most *BpMT*s (Fig. S1, 6). We hypothesize that the potential mechanisms of *BpMTs* in responding to HM stress relate to root growth and regulation, involving co-expression and interaction within the family. Future research on *B. papyrifera*’s response to HM stress will explore these potential pathways, including root control and ABA signaling.

## Materials and methods

### Plant materials and treatments

*B. papyrifera* seeds were collected from a mine tailing area in Xiangtan, Hunan Province (112°45′E, 27°53′N) and identified by Prof. Zhao Yunlin, with permission (Grant No. 2016TP2007). The seeds were germinated in a greenhouse (relative humidity 70 ± 5%, temperature 22 ± 2 °C, light/dark cycle 14/10 h) [34]. Seedlings were selected based on comprehensive evaluations of growth and resistance to HMs. Specimens from selected plants were preserved at the Plant Factory of the Forestry College of Northwest A&F University. Plant stem segments were used to establish a tissue culture system. Uniform tissue culture seedlings from the same subculture line (approximately 4 cm in height) were transplanted to pots after seven days of seedling acclimatization and maintained in a greenhouse. The potting mix consisted of soil, vermiculite, and perlite in a 5:3:2 ratio. After 10 months of growth with regular tap water irrigation, the seedlings were subjected to HM stress and ABA treatment. Each treatment solution (0.2 mmol/L CdCl_2_, 0.5 mmol/L MnCl_2_, 0.6 mmol/L CuSO_4_, 0.2 mmol/L ZnCl_2_, and 30 µmol/L ABA) was applied in 1 L volumes in trays to ensure the plants remained stressed until the end of the sampling period. Roots, stems, and leaves were harvested at 0 (control), 6, 24, and 72 h and stored at − 80 °C for RNA isolation. Each treatment had three replicates, with each replicate containing five seedlings. Control seedlings were water irrigated synchronously in equal amounts.

### Identification of *MT* genes in *B. papyrifera*

The root and leaf transcriptomes of *B. papyrifera*, subjected to treatments with 0.2 mmol/L CdCl_2_, 0.5 mmol/L MnCl_2_, 0.6 mmol/L CuSO_4_, 0.2 mmol/L ZnCl_2_, and 30 µmol/L ABA for 72 h, were sequenced to obtain a preliminary sequence set. The transcriptomes were assembled using Trinity. Gene functions were annotated against databases such as Nr, Nt, Pfam, KOG/COG, Swiss-Prot, KEGG, and GO [34]. Gene expression levels were estimated with RNA-seq by expectation maximization. Differential expression analyses under these conditions were conducted using the DESeq R package (version 1.10.1). Genes identified as differentially expressed by DESeq, with an adjusted P-value < 0.05, were considered for further analysis. The differentially expressed genes were screened for potential *MT* family candidates using a keyword search. Candidates were further validated through Basic Local Alignment Search Tool (nih.gov). The ORFs of potential *MT* genes were analyzed using ORF Finder (https://www.ncbi.nlm.nih.gov/orffinder/). Conserved domains were predicted using the online Conserved Domain Search Service (https://www.ncbi.nlm.nih.gov/Structure/cdd/wrpsb.cgi). The MT domain of all potential *BpMT*s was confirmed using the Hidden Markov Model by searching PFAM (http://pfam.sanger.ac.uk/search) and HMMER (https://www.ebi.ac.uk/tools/hmmer/search/phmmer) [35]. The conserved motifs were analyzed using the MEME online tool (http://alternate.meme-suite.org/) and Tbtools [36] with parameters set to a maximum of 10 motifs, allowing any number of repetitions, and a motif width range of 6–29. Following these analyses, the members of the *BpMT* family were confirmed. Cloning primers for the ORFs were designed based on cDNA and subsequently verified by sequencing. The MW, pI, and amino acid composition of the confirmed *BpMTs* were analyzed using the ExPASy server (https://web.expasy.org/protparam/).

### Analysis of evolutionary relationship of BpMTs

To elucidate the evolutionary relationships of the *B. papyrifera MT* genes, MT protein sequences from *G. max*, *O. sativa*, *Z. mays*, *(A) thaliana*, *V. vinifera*, and *M. pumila* were downloaded from the database Phytozome v13 (https://phytozome-next.jgi.doe.gov/). The MT protein sequences from *(B) papyrifera* and these species were aligned using the Clustal W2 program. A phylogenetic tree was constructed using MEGA7 via the neighbor-joining method [37]. The evolutionary tree was visualized using the online software ITOL (https://itol.embl.de/) [38]. The BpMTs were classified into subclasses based on the topology of the phylogenetic tree.

### Expression analysis of *BpMTs* under HM stress and ABA

Total RNA was extracted from each sample using the cetyltrimethylammonium bromide method [34]. The quality of the RNA was verified by 1% agarose gel electrophoresis, and its concentration was determined before reverse transcription, following digestion with DNase I (Takara, Dalian, China). Reverse transcription of RNA to cDNA was performed using the PrimeScript™ RT reagent Kit (CWBIO, Beijing, China). The resulting cDNA was diluted 10 times and used as a template for quantitative real-time PCR (qRT-PCR) analysis. qRT-PCR was carried out using SYBR Green Real-Time PCR Master Mix (CWBIO) on the StepOne™ Real-Time PCR system (Applied Biosystems). The reaction protocol included an initial denaturation at 94 °C for 30 s; 45 cycles of denaturation at 94 °C for 12 s, annealing at 60 °C for 45 s, and extension at 72 °C for 45 s; followed by a final melt curve step at 81 °C for 1 s. To ensure reproducibility, each qRT-PCR was performed in triplicate for each sample. Two *B. papyrifera* genes, *actin* [39] and *GAPDH* [40], served as internal reference genes. The quantitative results were analyzed using the 2^–ΔΔCT^ method [41], and the relative expression levels were normalized to the average value of the two internal reference genes. All primers used for qRT-PCR are listed in Table S1.

### HM stress response potential analysis in yeast

The ORFs of the *BpMTs* were amplified by PCR using primers containing specific restriction endonuclease cleavage sites (Table S2). The PCR-amplified products were digested with the corresponding enzymes and cloned into the yeast expression vector pYES2 to construct recombinant vectors pYES2-BpMTs. Both pYES2-BpMT and empty pYES2 were independently transformed into *Saccharomyces cerevisiae* INVSC1 (His, Leu, Trp, and Ura) using the lithium acetate method [42]. INVSC1 yeast colonies harboring empty pYES2 (control) and pYES2-BpMTs were designated as INVSC1(pYES2) and INVSC1(pYES2-BpMT1a) to INVSC1(pYES2-BpMT3a), respectively. These were cultivated in Sc-Ura medium containing 2% glucose at 30 °C to an OD_600_ of 0.6. Subsequently, the yeast was diluted in Sc-Ura medium including 2% galactose and maintained at 30 °C for approximately 20 h before re-collecting and adjusting the OD_600_ of the yeast to 1.8 for stress analysis.

For HM stress testing, the collected INVSC1(pYES2) and INVSC1(pYES2-BpMTs) were individually treated with 0.6 mmol/L CdCl_2_, 0.5 mmol/L MnCl_2_, 0.6 mmol/L CuSO_4_, and 0.2 mmol/L ZnCl_2_ for 6 h, followed by serial dilutions spotted onto Sc-Ura agar plates and incubated at 30 °C for about 48 h. To quantitatively compare survival rates under these stresses, OD_600_ values were tested and compared under both normal and stress conditions. Specifically, 5 mL yeast cultures starting at an OD_600_ of 1.8 were incubated with the listed HMs for 6 h. A 1 mL sample from these 5 mL cultures was then added to 30 mL of fresh lipid medium and incubated at 30 °C for 24 h with shaking at 185 rpm, after which cell densities (OD_600_) were measured. Each stress test was conducted in triplicate.

### Mutation of Cys residues and HM tolerance analysis in yeast

The Cys (TGT and TGC) residues at the N-termini and C-termini of *BpMT1c*, *BpMT2d*, and *BpMT2e*, numbering 6, 8, and 8 at the N-termini and 6, 6, and 6 at the C-termini respectively, were mutated to Ser (AGC). The mutated N-terminal sequences were labeled as BpMT1c-mΔN, BpMT2d-mΔN, and BpMT2e-mΔN, and the C-terminal mutated genes as BpMT1c-mΔC, BpMT2d-mΔC, and BpMT2e-mΔC. These mutations were all PCR cloned using specific mutated primers (Table S2) based on the plasmid of pYESE-BpMT1c, pYESE-BpMT2d, and pYESE-BpMT2e, respectively, following a protocol of 37 °C for 20 min; 94 °C for 3 min; 35 cycles at 94 °C for 30 s, 65 °C for 30 s, 72 °C for 30 s; and a final extension at 72 °C for 7 min. The mutated sequences were then independently inserted into pYES2 to generate mutated yeast expression recombinants pYES2-BpMT1c-mΔN, pYES2-BpMT2d-mΔN, pYES2-BpMT2e-mΔN, pYES2-BpMT1c-mΔC, pYES2-BpMT2d-mΔC, and pYES2-BpMT2e-mΔC. Each recombinant protein was transfected into INVSC1 cells using the aforementioned method. The Cd, Cu, Mn, and Zn tolerance assays were conducted similarly to those for INVSC1(pYES2-BpMT1a) to INVSC1(pYES2-BpMT3a).

### Quantification of HM accumulation by ICP-MS

To quantify the accumulation of HMs (Cd, Cu, Mn, and Zn), yeast strains harboring pYES2, pYES2-BpMT1c, pYES2-BpMT1c-mΔN, pYES2-BpMT1c-mΔC, pYES2-BpMT2d, pYES2-BpMT2d-mΔN, pYES2-BpMT2d-mΔC, pYES2-BpMT2e, pYES2-BpMT2e-mΔN, and pYES2-BpMT2e-mΔC were washed three times with sterilized ddH_2_O. The washed yeasts were then dried in a hot air oven at 40 °C. The dried yeast was dissolved in 6 mL of HNO_3_ and 2 mL of H_2_O_2_ within a microwave digester. The final volumes of the digested samples were adjusted to 10 mL, filtered to remove any impurities, and analyzed for Cd, Cu, Mn, and Zn using ICP-MS [43].

### Statistical analysis

The data were processed using the SPSS package (SPSS, Chicago, Illinois, USA). Standard deviation means the sample variability. Expression differences between different time points and 0 d were evaluated by T test (*P* < 0.05). The differences among different yeast strains were confirmed using ANOVA analysis (*P* < 0.05). Some results were visualized in Tbtools software and Origin 2017 and showed as heat maps[46].

## Conclusions

In this study, the *MT* family genes were analyzed within the *B. papyrifera* transcriptome, resulting in the identification of 12 *BpMT* genes. These genes are highly conserved and categorized into three types: MT1, MT2, and MT3. Each member of this family shares similar structures and conserved motifs. All *BpMT* genes were induced by Cd, Cu, Mn, and Zn stress, displaying various expression levels and demonstrating a positive role in the HM stress response via the ABA-dependent signaling pathway. There were significant correlations among *BpMT* family members in response to Cd, Cu, Mn, and Zn stress, suggesting that *BpMT* family genes may respond to HM stress through co-expression or interactions within the family. Furthermore, Cys residue mutation assays revealed that type 1 *BpMT1c* primarily achieved HM tolerance due to its C-terminal conserved domain, while type 2 *BpMT2d* and *BpMT2e* exhibited similar functions in both the N- and C-terminal domains. These findings provide novel insights into the *MT* family genes related to paper mulberry’s HM tolerance and chelation capabilities.

### Electronic supplementary material

Below is the link to the electronic supplementary material.

Supplementary Material 1

## Data Availability

All the data were presented in the main manuscript and additional supporting files. The Arabidopsis related datasets generated and/or analysed during the current study are available in the TAIR database (https://www.arabidopsis.org/). The MT protein sequences of *G. max*, *O. sativa*, *Zea mays*, *A. thaliana*, *V. vinifera and M. pumila* were downloaded from the database phytozome13v (https://phytozome-next.jgi.doe.gov/).
