# Supplementary material for: Characterization of metallothionein genes from Broussonetia papyrifera: metal binding and heavy metal tolerance mechanisms
Source: BMC Genomics. 2024 Jun 5;25:563. doi: 10.1186/s12864-024-10477-x (PMC11151532; doi:10.1186/s12864-024-10477-x)
Supplement: Supplementary file 1 — Supplementary Material 1 [file 12864_2024_10477_MOESM1_ESM.doc]

**Table S1 The primers used for qRT-PCR analysis.**

| Primer name | Forward primer | Reverse primer |
| --- | --- | --- |
| BpMT1a-F/R | 5′-ATGTCTTGCAACTGTGG-3′ | 5′- AGTTGCAAGGGTTGCACT-3′ |
| BpMT1b-F/R | 5′-ATGTCTTGCAACTGCGGATC-3′ | 5′-CAGTTGCAAGGGTTGCACTT-3′ |
| BpMT1c-F/R | 5′-TGTCTTGCAGCTGTGGAT-3′ | 5′-AACAGTTGCAAGGGTTGC-3′ |
| BpMT1d-F/R | 5′-ATGTCTTGCAGCTGCGGAT-3′ | 5′-TTGCACTTGCAGTTGTCACC-3′ |
| BpMT2a-F/R | 5′-ATGTCGTGCTGTGGTGGCAACT-3′ | 5′-CACTTGCAGGTGCACGGGTTGC-3′ |
| BpMT2b-F/R | 5′-GAAGATGTCTTGCTGTGG-3′ | 5′-TTACAGGTGCATGGGTTG-3′ |
| BpMT2c-F/R | 5′-ATGTCTTGCTGTGGTGGA-3′ | 5′-ACAGTTGCATGGGTTGC -3′ |
| BpMT2d-F/R | 5′-GCAAGATGTACCCTGACT-3′ | 5′-CAAGTGTGGCTTCTCAGT-3′ |
| BpMT2e-F/R | 5′-ATGTCCTGCTGCGGAGG-3′ | 5′-TCACTTGCAGGTGCAGG-3′ |
| BpMT2f-F/R | 5′-ATGTCTTGCTGCGGAGGAAACT -3′ | 5′-TCATTTGCAGGTGCAAGGGTC-3′ |
| BpMT2g-F/R | 5′-ATGTCTTGCTGCGGAGGA-3′ | 5′-CTGCAGTTGGCTCCACAT-3′ |
| BpMT3a-F/R | 5′-ATGGATCCCAACTGCTCCT-3′ | 5′-TCAGGCGCAGCAGCTGCAC-3′ |
| BpActin-F/R | 5′-CAATCCAAGAGAGGTATCC-3′ | 5′-CATTGCTGGAGTGTTGAAGG-3′ |
| BpGAPDH-F/R | 5′-TCAACATCATTCCTAGCAGTACCG-3′ | 5′-AGTCAGTGGAAACCACGTCATC-3′ |

**Table S2 The primers used for constructing the yeast expression recombinant vectors.**

| Primer name | Forward primer (5′- -3′) | Reverse primer (5′- -3′) |
| --- | --- | --- |
| BpMT1a-JM | ATCGGGTACCATGTCTTGCAACTGTGGATC | CGATTCTAGATTAGCAGTTGCAAGGGTTGC |
| BpMT1b-JM | ATCGGGTACCATGTCTTGCAACTGCGGATC | CGATTCTAGATTAGCAGTTGCAAGGGTTGC |
| BpMT1c-JM | ATCGGGTACCATGTCTTGCAGCTGTGGATC | CGATTCTAGATTAACAGTTGCAAGGGTTGC |
| BpMT1d-JM | ATCGGGTACCATGTCTTGCAGCTGCGGAT | CGATTCTAGATCAGCAGTTGCAGGGGTTGC |
| BpMT2a-JM | ATCGGGTACCATGTCGTGCTGTGGTGGC | CGATTCTAGATCACTTGCAGGTGCACGG |
| BpMT2b-JM | ATCGGGTACCATGTCCTGCTGCGGAGG | CGATTCTAGATCATTTACAGGTGCATGGGTT |
| BpMT2c-JM | ATCGGGTACCATGTCTTGCTGTGGTGGA | CGATTCTAGATCATTTACAGTTGCATGG |
| BpMT2d-JM | ATCGGGTACCATGTCTTGCTGTGGTGG | CGATTCTAGATCATTTACAGTTGCATG |
| BpMT2e-JM | ATCGGGTACCATGTCCTGCTGCGGAGG | CGATTCTAGATCACTTGCAGGTGCAGG |
| BpMT2f-JM | ATCGGGTACCATGTCTTGCTGCGGAGGAAACT | CGATTCTAGATCATTTGCAGGTGCAAGGGTC |
| BpMT2g-JM | ATCGGGTACCATGTCTTGCTGCGGAGGA | CGATTCTAGATCATTTGCAAGTGCAAGG |
| BpMT3a-JM | ATCGGGTACCATGGATCCCAACTGCTCCT | CGATTCTAGATCAGGCGCAGCAGCTGCAC |
| BpMT1c-mΔN-JM | ATCGGGTACCATGTCTAGCAGCAGTGGATCTAGCAGCAGCAGCGGCTCAAACAGCTCCAGC | CGATTCTAGATTAACAGTTGCAAGGGTTGCACCTGCAGCTGGAACCGCAGCTGCAGCCATG |
| BpMT1c-mΔC-JM | ATCGGGTACCATGTCTTGCAGCTGTGGATCTAGCTGCAGCTGCGGCTCAAACTGC | CGATTCTAGATTAACTGTTGCTAGGGTTGCTCCTGCTGCTGGAACCGCTGCTGCT |
| BpMT2d-mΔN-JM | ATCGGGTACCATGTCTAGCAGTGGTGGAAACAGTGGCAGCGGCTCTGGCAGCAAGAGCGGCACTGGCAGTGGAGGGAGC | CGATTCTAGATCATTTACAGTTGCATGGGTTGCACGTGCAGTTGGCTCCACACTTGCACCCGTTCTCTGCC |
| BpMT2d-mΔC-JM | ATCGGGTACCATGTCTTGCTGTGGTGGAAACTGTGGCTGCGGCTCTGGCTGCAAGTGC | CGATTCTAGATCATTTACTGTTGCTTGGGTTGCTCGTGCTGTTGGCTCCACTCTTGCT |
| BpMT2e-mΔN-JM | ATCGGGTACCATGTCCAGCAGCGGAGGCAACAGCGGGAGCGGCTCCGGCAGCAAGAGCGGCAGCGGCAGCGGAGGGAGC | CGATTCTAGATCACTTGCAGGTGCAGGGGTTGCAGCTGCAGTTGGGGCCGCACTTGCAACCGTCGTTCTCAGCTC |
| BpMT2e-mΔC-JM | ATCGGGTACCATGTCCTGCTGCGGAGGCAACTGCGGGTGCGGCTCCGGCTGCAAGTG | CGATTCTAGATCACTTGCTGGTGCTGGGGTTGCTGCTGCTGTTGGGGCCGCTCTTGCT |


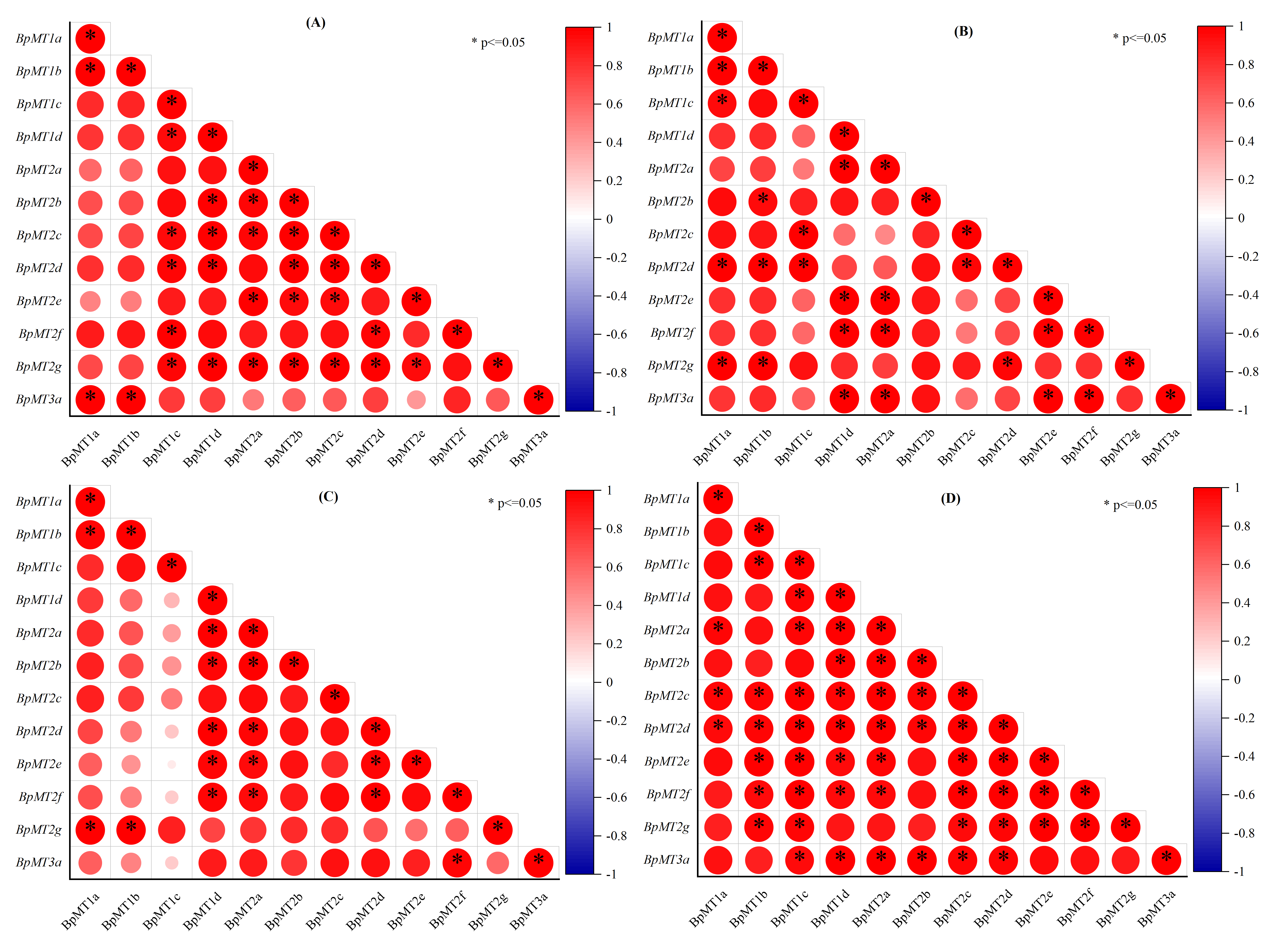


**Fig. S1** Correlation analysis between *BpMT* genes under different treatments. Red indicates positive correlation, while blue indicates negative correlation. * Indicates the correlation is significant (*P*<0.05). (A), Cd; (B), Cu; (C), Mn; (D), Zn.
